# Assessment of the proposed EBMT pediatric criteria for diagnosis and severity grading of sinusoidal obstruction syndrome

**DOI:** 10.1038/s41409-018-0426-8

**Published:** 2019-01-25

**Authors:** Marte B. Kammersgaard, Katrine Kielsen, Carsten Heilmann, Marianne Ifversen, Klaus Müller

**Affiliations:** 1 Institute for Inflammation Research, Department of Rheumatology and Spine Disease, Copenhagen University Hospital, Rigshospitalet, Copenhagen, Denmark; 2 Department of Pediatrics and Adolescent Medicine, Copenhagen University Hospital, Rigshospitalet, Copenhagen, Denmark

**Keywords:** Diagnosis, Paediatrics

## Abstract

Sinusoidal obstruction syndrome (SOS) is a potentially life-threatening complication of allogeneic hematopoietic stem cell transplantation (HSCT). We assessed the proposed pediatric EBMT criteria along with the Baltimore and modified Seattle criteria in a population-based cohort. Eighty-seven children (1.1–17.3 years) undergoing myeloablative HSCT from 2010 to 2017 were consecutively included at the Danish National Transplantation Center. In total, 39 (44.8%) patients fulfilled the EBMT criteria and 30 patients (35%) fulfilled the criteria for severe or very severe SOS. Nine (10.3%) patients fulfilled the modified Seattle criteria while none met the Baltimore criteria. Patients fulfilling the EBMT criteria for SOS had longer primary admission (31 days (23–183) vs. 27 days (17–61), *p* = 0.001), were treated more intensively with diuretics within the first 3 months (29 days (0–90) vs. 3.5 days (0–90), *p* < 0.0001), and had a longer time to stable platelet counts >50 × 10^9^/L (32 days (16–183) vs. 23 days (14–101), *p* < 0.0001). Two patients, fulfilling neither Baltimore nor Seattle criteria, but selectively fulfilling EBMT criteria, died of treatment-related acute inflammatory complications within 1 year post-HSCT. In conclusion, application of the pediatric EBMT diagnostic and severity criteria may be helpful in identifying patients at increased risk of severe treatment-related complications and mortality, although with a risk of over-diagnosing SOS.

## Introduction

Sinusoidal obstruction syndrome (SOS), also known as veno-occlusive disease (VOD; hereafter referred to as SOS), is a potentially life-threatening complication of allogeneic hematopoietic stem cell transplantation (HSCT) [1]. The initiating pathogenic step is damage to sinusoidal endothelial cells in the hepatic acinus, initiated by toxic metabolites of the conditioning regimen. This may lead to vascular occlusion, capillary leakage and hepatocellular necrosis, causing fluid overload, consumptive and transfusion-refractory thrombocytopenia and hyperbilirubinemia [2–4]. Most cases resolve within weeks, but a variable percentage of up to 30–60% have been reported to progress to multi-organ dysfunction/failure (MOD/MOF) with a mortality rate of >80% [3, 5–7].

The reported incidence of SOS in children is variable and partly related to the diagnostic criteria applied, reportedly ranging from 20% to 60% in high-risk populations after allogeneic HSCT [5, 7–10]. Transplant-related risk factors include second myeloablative transplantation, unrelated and HLA-mismatched donor, high-dose or unfractionated total body irradiation (TBI) and conditioning with high-dose busulfan and cyclophosphamide [4, 9–11]. Furthermore, acute graft-versus-host disease (GvHD) and previous hepatic disease are risk factors [4, 10, 12]. In addition, a number of pediatric factors such as young age and low weight as well as certain genetic diseases are associated with increased incidence of SOS [4, 5, 7, 9, 10, 13].

Until recently, SOS has been diagnosed using the Seattle [14] and Baltimore [15] criteria in both children and adults, with modification of the Seattle criteria to require a 5% weight gain in children [5]. However, the use of similar criteria for children and adults is challenged by age-related differences in the clinical presentation. In 15–20% of cases, children present with SOS later than a month after HSCT [7, 9], which is rare in adults [16], and while hyperbilirubinemia is an indispensable requirement in the Baltimore criteria, anicteric SOS has been reported in about one-third of children, including those experiencing severe SOS [5]. If hyperbilirubinemia is present at an early stage, it is often pre-existing, caused by the primary diagnosis, or it may occur late in a severe case of SOS [7, 17, 18].

Accordingly, pediatric diagnostic criteria for SOS have been suggested by a working group under the European Society for Blood and Marrow Transplantation (EBMT). In these new criteria, the time-restriction of the Seattle and Baltimore criteria has been omitted, and hyperbilirubinemia and weight gain are evaluated based on individual baselines, taking pre-existing clinical conditions into account. To avoid potentially misleading insignificant changes, weight gain and increase in bilirubin are assessed over 3 consecutive days and imaging techniques for ascites and hepatomegaly are recommended to improve sensitivity and specificity of the criteria [7]. Finally, transfusion refractory thrombocytopenia (RT) has been added as a criterion (Table 2) [16, 19–21].

Clinical studies indicate that defibrotide is effective for treatment of SOS in children [5, 6, 22, 23], especially after early intervention [24, 25], underlining the need for early diagnosis of SOS. Since the proposed pediatric SOS criteria are based on expert opinion like the Seattle and Baltimore criteria, empirical studies are needed to assess their validity and their applicability in the clinic. The Seattle and Baltimore criteria have a reported specificity of 95% and 89%, respectively [14, 15], with a low sensitivity of 56% [26], though this is mainly based on studies in adult HSCT which cannot directly be applied to children due to differences in clinical presentation of SOS.

The purpose of this retrospective study was to assess the new pediatric EBMT diagnostic criteria and severity grading along with the classical Baltimore and modified Seattle criteria in a clinical study.

## Patients and methods

### Study population

In this population-based study, 87 children (1–18 years of age) undergoing allogeneic HSCT were consecutively recruited at Copenhagen University Hospital Rigshospitalet, Denmark, from June 2010 to December 2012 and from March 2015 to June 2017, for studies of toxicities and immune reconstitution as described previously [27–30]. One patient was excluded due to death from fungal infection on day +9 without signs of SOS. Written informed consent was obtained from all included patients and/or their legal guardians after approval by the local ethics committee (H-1-2010-009 and H-7-2014-016). The patients were followed for 1 year post-transplant with an average follow-up time of 314 days (56–365). Thirteen patients did not complete a full year of follow-up due to relapse (*n* = 6), graft rejection (*n* = 4), or treatment-related death (*n* = 3).

The clinical characteristics are listed in Table 1. Diagnoses were malignant (*n* = 51) or benign diseases (*n* = 36). Donors were either matched siblings (MSD) (*n* = 22), matched unrelated donors (MUD) (*n* = 41), mismatched unrelated donors (MMUD) (*n* = 10), haploidentical (*n* = 6), or umbilical cord blood (UCB) (*n* = 8). Stem cell sources were bone marrow (BM) (*n* = 69), peripheral blood stem cells (PBSC) (*n* = 10), or UCB grafts (*n* = 8). Conditioning regimens were TBI-based (*n* = 21), busulfan-based (*n* = 42), or other chemotherapy-based regimens (*n* = 24).

**Table 1:** Patient and transplant characteristics

Patient and transplant characteristics	*n* = 87
Males	49 (56.3%)
Age at transplantation (years), median (range)	
Recipients	7.8 (1.1–17.3)
Donors	22.9 (0.0–58.4)
Disease at transplantation, no. (%)	
Acute lymphoblastic leukemia	27 (31.0%)
Acute myeloid leukemia	11 (12.6%)
Myelodysplastic syndrome	5 (5.7%)
Other malignancies	8 (9.2%)
Severe aplastic anemia	7 (8.0%)
Thalassemia	3 (3.4%)
Hemophagocytic lymphohistiocytosis	2 (2.3%)
X-linked lymphoproliferative syndrome	2 (2.3%)
Pediatric immunodeficiency syndromes	12 (13.8%)
Infantile osteopetrosis	1 (1.1%)
Other benign disorders	9 (10.3%)
Donor type, no. (%)	
Matched sibling donor (10/10)	22 (25.3%)
Matched unrelated donor (10/10)	41 (47.1%)
Mismatched unrelated donor (9/10)	10 (11.5%)
Umbilical cord blood (8/10)	8 (9.2%)
Haploidentical donor	6 (6.9%)
Stem cell source, no. (%)	
Bone marrow	69 (79.3%)
Peripheral blood stem cells	10 (11.5%)
Umbilical cord blood	8 (9.2%)
Conditioning regime, no. (%)	
TBI (1200 cGy) + VP16 or CY	17 (19.5%)
TBI (200 cGy) + CY	4 (4.6%)
BU + CY ± VP16	10 (11.5%)
BU + CY + MEL	15 (17.2%)
BU + other	17 (19.5%)
Other chemotherapy-based conditioning	24 (27.6%)
ATG as part of conditioning regimen, no. (%)	67 (77.0%)
Ciclosporin as GvHD prophylaxis, no. (%)	79 (90.8%)
HSCT no.	
1	85 (97.7%)
2	2 (2.3%)
Baseline bilirubin, median (range)	5.6 (2.2–43.0)
High risk of SOS, no. (%)	25 (28.7%)
Defibrotide prophylaxis, no. (%)	5 (5.7%)

*TBI* total body irradiation, *BU* busulfan, *CY* cyclophosphamide, *MEL* melphalan, *VP16* etoposide, *ATG* anti-thymocyte globulin, *GvHD* graft-versus-host disease, *HSCT* hematopoietic stem cell transplantation

Four patients (4.6%) had a baseline bilirubin above normal range. Twenty-five patients (28.7%) had a high risk of developing SOS due to prior HSCT, allogeneic HSCT for leukemia beyond the second relapse, diagnoses of adrenoleukodystrophy, osteopetrosis or macrophage activation syndrome or conditioning with busulfan and melphalan, while no patients presented with pre-existing liver disease or received ozogamicin-coupled monoclonal antibodies (gemtuzumab or ozogamicin) [5]. Defibrotide was given as SOS-prophylaxis to certain high-risk patients by the clinician in charge based on a general clinical assessment, and most frequently after the approval in 2016.

### Assessment of criteria

Clinical parameters were retrospectively registered from the patient’s medical records for the first year following HSCT. The applied pediatric EBMT criteria are stated in Table 2.

**Table 2:** Criteria for the diagnosis of SOS

Modified Seattle criteria^a^	Baltimore criteria	EBMT pediatric criteria
**Presence before day 20 after HSCT** ≥2 of the following:	**Presence before day 21 after HSCT** Bilirubin criterion plus ≥2 of the other criteria	**No limitation for time of onset** ≥2 of the following:
Bilirubin ≥34 μmol/L	Bilirubin ≥34 μmol/L	Bilirubin ≥34 µmol/L within 72 h or rising bilirubin from a baseline value on 3 consecutive days
Weight gain >5% from baseline	Weight gain >5% from baseline	A weight gain >5% above baseline value or otherwise unexplained weight gain on 3 consecutive days despite the use of diuretics
Hepatomegaly or right upper quadrant pain	Hepatomegaly (usually painful)	Hepatomegaly (best if confirmed by imaging) above baseline value^b^
	Ascites	Ascites (best if confirmed by imaging) above baseline value^b^
		Unexplained consumptive and transfusion-refractory thrombocytopenia^c^

*HSCT* hematopoietic stem cell transplantation

All of the criteria above demand the exclusion of differential diagnoses [7, 14, 15]

This table is adapted from the respectable journals [7, 14, 15]

^a^Seattle criteria are modified in children to demand a weight gain >5% from baseline instead of >2%

^b^Suggested: imaging (ultrasonography, computed tomography, or magnetic resonance imaging) immediately before HSCT to determine baseline value for both hepatomegaly and ascites

^c^≥1 Weight-adjusted platelet substitution/day to maintain institutional transfusion guidelines

Some of the EBMT criteria required supplementary specifications not detailed in the article by Corbacioglu et al. [7]. In the present study, bilirubin was considered increased if either above normal range for the patient's age and sex or if higher than 4 times the baseline value, as this combination appeared to result in a consistent assessment of rise in bilirubin. Further, baseline bilirubin was defined as the average of the last 2–3 values measured prior to conditioning. Refractory consumptive RT was defined as the need for otherwise unexplained platelet transfusions daily for ≥3 days to keep platelet counts above transfusion levels (20 × 10^9^/L). Bilirubin and platelet counts were measured at least once daily as a routine procedure during hospitalization, and patients were weighed at least once daily during the admission to monitor hydration. For patients fulfilling EBMT criteria at more than one occasion, only data related to the first time point of SOS were applied in this analysis.

In this study, patients were severity graded for maximum grade of SOS by applying the pediatric EBMT severity grading criteria [7]. These criteria categorize SOS as mild, moderate, severe, or very severe (grade I–IV) based on the extent of the following parameters: duration of persistent RT, rise of liver biomarkers, rise and kinetics of bilirubin, amount of ascites, and impaired coagulation as well as signs of renal, pulmonary, or CNS organ dysfunction [7]. Of liver transaminases, only alanine aminotransferase (ALT) was available in all patients. International Normalized Ratio and coagulation factors II, VII, and X (both measured with ACL TOP), as well as need for fresh frozen plasma, were used to evaluate impaired coagulation. Oxygen requirement and new onset cognitive impairment were registered from medical records to assess pulmonary and CNS dysfunction, respectively. Due to scarce data on estimated glomerular filtration rate (eGFR) based on EDTA clearance, eGFR was calculated based on cystatin C (*n* = 31) [31] or creatinine (*n* = 8) [32]. Creatinine was measured at least daily during hospitalization (with Cobas 8000 c702), and Cystatin C was measured when indicated by the clinical condition and at least weekly for most of the period from August 2012 (with Cobas 8000 c502).

### Statistical analyses

The Mann–Whitney-*U* test, Wilcoxon rank sum test, or Kruskal–Wallis rank sum test were used to calculate differences between continuous variables. Fisher’s exact test was used for categorical variables.

Kaplan–Meier estimates with log-rank test were applied for overall survival, transplant-related mortality, relapse, duration of primary admission, admission to the intensive care unit (ICU), acute and chronic GvHD, time to neutrophil engraftment, and time to stable platelet counts >50 × 10^9^/L.

A two-sided *p*-value < 0.05 was considered statistically significant. All statistical analyses were performed using R statistical software version 3.4.2 (The R Foundation for Statistical Computing, Vienna, Austria) and R studio (R Studio, Boston, MA, USA).

## Results

### Incidence of SOS

**Fig. 1:**
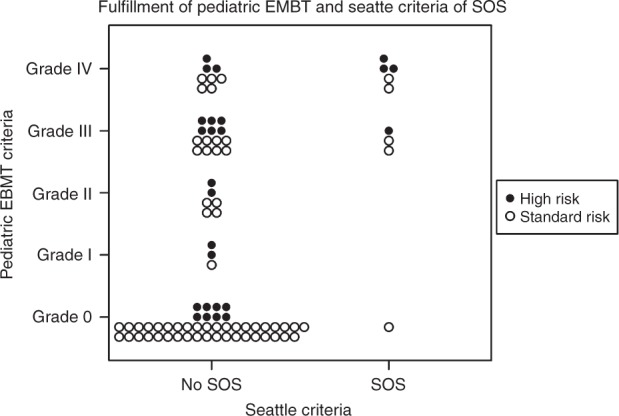
Comparison of the diagnostic criteria for patients with high- and standard-risk of developing SOS. A child transplanted with a myeloablative conditioning is considered to be in high risk of SOS in case of second myeloablative stem cell transplant, allogeneic stem cell transplants for leukemia >2 relapses, liver disease, history of treatment with ozogamicin-coupled monoclonal antibodies, conditioning with busulfan and melphalan, osteopetrosis, macrophage activating syndromes or adrenoleukodystrophy [5]

Thirty-nine (44.8%) patients fulfilled the EBMT criteria, while 9 (10.3%) patients fulfilled the modified Seattle criteria. Of the patients fulfilling the Seattle criteria, 8 out of 9 also fulfilled the EBMT criteria (Fig. 1), while one patient only fulfilled the modified Seattle criteria with upper right quadrant pain and bilirubin ≥34 µmol/L. None of the patients met the Baltimore criteria. Three patients were treated with defibrotide: one fulfilling Seattle criteria, one on suspicion of late onset SOS, and one with suspected pulmonary VOD.

Median time to diagnosis was 6 days from HSCT (0–54 days) with EBMT criteria, and 6 days (1–13 days) with Seattle criteria. For the 8 patients fulfilling both Seattle and EBMT criteria, EBMT criteria were in average fulfilled 3 days earlier than Seattle criteria. Patients fulfilling the EBMT criteria presented with rising bilirubin (*n* = 33, 84.6%), consumptive RT (*n* = 31, 79.5%), weight gain (*n* = 26, 66.7%), ultrasound-confirmed hepatomegaly (*n* = 1, 2.6%), and ultrasound-confirmed ascites (*n* = 1, 2.6%). Four patients fulfilled EBMT criteria at two separate time points.

When grading the patients according to the pediatric EBMT severity criteria, 13 (14.9%) were classified as grade IV, 17 (19.5%) as grade III, 6 (6.9%) as grade II, and 3 (3.4%) as grade I SOS.

### Patient characteristics and development of SOS

Grade III–IV SOS was associated with malignant diagnoses and conditioning with busulfan plus cyclophosphamide (*p* = 0.039 and *p* = 0.015, respectively). There were no associations with recipient age, donor type, stem cell source, conditioning with TBI, or baseline levels of bilirubin (Tables 3 and 4). Patients with malignant diseases received TBI-based conditioning or conditioning with cyclophosphamide plus busulfan plus/minus etoposide or melphalan more commonly than patients with benign diseases (*p* < 0.0001 and *p* = 0.0006, respectively).

**Table 3:** Patient and transplant characteristics according to fulfillment of pediatric EBMT diagnostic and severity grading criteria for SOS

Patient and transplant characteristics	Grade 0	Grade I–II	Grade III–IV
Total number of patients, no. (%)	48 (55.2%)	9 (10.3%)	30 (34.5%)
Males	28 (57.1%)	5 (10.2%)	16 (32.7%)
Age at transplantation (years), median (range)			
Recipients	7.5 (1.1–16.6)	3.4 (1.2–13.4)	9.1 (1.2–17.3)
Donors	23.1 (0.0–58.4)	22.6 (5.5–45.4)	20.2 (0.0–51.4)
Disease at transplantation, no. (%)			
Acute lymphoblastic leukemia	11 (40.7%)	3 (11.1%)	13 (48.1%)
Acute myeloid leukemia	3 (27.3%)	1 (9.1%)	7 (63.6%)
Myelodysplastic syndrome	4 (80.0%)	0 (0.0%)	1 (20.0%)
Other malignancies	5 (62.5%)	1 (12.5%)	2 (25.0%)
Severe aplastic anemia	6 (85.7%)	0 (0.0%)	1 (14.3%)
Thalassemia	3 (100.0%)	0 (0.0%)	0 (0.0%)
Hemophagocytic lymphohistiocytosis	1 (50.0%)	0 (0.0%)	1 (50.0%)
X-linked lymphoproliferative syndrome	2 (100%)	0 (0.0%)	0 (0.0%)
Pediatric immunodeficiency syndromes	8 (66.7%)	3 (25%)	1 (8.3%)
Infantile osteopetrosis	0 (0.0%)	0 (0.0%)	1 (100.0%)
Other benign disorders	5 (55.6%)	1 (11.1%)	3 (33.3%)
Donor type, no. (%)			
Matched sibling donor (10/10)	10 (45.5%)	4 (18.2%)	8 (36.4%)
Matched unrelated donor (10/10)	25 (61.0%)	3 (7.3%)	13 (31.7%)
Mismatched unrelated donor (9/10)	5 (50.0%)	1 (10.0%)	4 (40.0%)
Umbilical cord blood (8/10)	4 (50.0%)	0 (0.0%)	4 (50.0%)
Haploidentical donor	4 (66.7%)	1 (16.7%)	1 (16.7%)
Stem cell source			
Bone marrow	39 (56.5%)	8 (11.6%)	22 (31.9%)
Peripheral blood stem cells	5 (50.0%)	1 (10.0%)	4 (40.0%)
Umbilical cord blood	4 (50.0%)	0 (0.0%)	4 (50.0%)
Conditioning regime, no. (%)			
TBI (1200 cGy) + VP16 or CY	10 (58.8%)	1 (5.9%)	6 (35.3%)
TBI (200 cGy) + CY	4 (100.0%)	0 (0.0%)	0 (0.0%)
BU + CY ± VP16	3 (30.0%)	1 (10.0%)	6 (60.0%)
BU + CY + MEL	5 (33.3%)	2 (13.3%)	8 (53.3%)
BU + other	8 (47.1%)	1 (5.9%)	8 (47.1%)
Other	18 (75%)	4 (16.7%)	2 (8.3%)
ATG as part of conditioning regimen, no. (%)	40 (59.7%)	5 (7.5%)	22 (32.8%)
Ciclosporin as GvHD prophylaxis, no. (%)	43 (54.4%)	7 (8.9%)	29 (36.7%)
HSCT, no. (%)			
1st	48 (56.5%)	7 (8.2%)	30 (35.3%)
2nd	0 (0.0%)	2 (100.0%)	0 (0.0%)
Baseline bilirubin, median (range)	5.5 (2.4–43.0)	3.7 (2.2–13.3)	6.0 (3.0–17.7)
High risk of SOS, no. (%)	8 (32%)	4 (16%)	13 (52%)
Defibrotide prophylaxis, no. (%)	2 (40.0%)	0 (0.0%)	3 (60.0%)

*TBI* total body irradiation, *BU* busulfan, *CY* cyclophosphamide, *MEL* melphalan, *VP16* etoposide, *ATG* anti-thymocyte globulin, *GvHD* graft-versus-host disease, *HSCT* hematopoietic stem cell transplantation

**Table 4:** Patient and transplant characteristics of patients diagnosed with severe SOS grade III–IV according to EBMT criteria

	Patient ID	Sex	Age at transplantation (years)	Diagnosis	Donor type	Stem cell source	HSCT, no.	Conditioning	High risk of SOS	Defibrotide prophylaxis pre-transplant (day)	Grade of EBMT SOS (day)	Modified Seattle SOS (day)	Baltimore SOS (day)	Defibrotide treatment (day)	Cause of death (day post-HSCT)
EBMT SOS grade III–IV	17	Male	11.5	Acute myeloid leukemia	SIB	BM	1	BU + CY + MEL	Yes	-	IV (+5)	Yes (+8)	-	-	-
	23	Male	13.7	Myelodysplastic syndrome	UCB	UCB	1	BU + CY + ATG	-	-	IV (0)	-	-	-	-
	24	Male	13.9	Acute myeloid leukemia	SIB	BM	1	BU + CY + MEL	Yes	-	IV (0)	Yes (+1)	-	-	-
	34	Male	12.6	Congenital sideroblastic anemia	MUD	BM	1	BU + CY + ATG	-	-	IV (+4)	Yes (+4)	-	-	-
	41	Male	3.4	Acute lymphoblastic leukemia	MMUD	BM	1	BU + THIO + FLU + ATG	-	-	IV (+4)	-	-	-	-
	44	Female	8.3	Acute myeloid leukemia	MUD	BM	1	BU + CY + MEL + ATG	Yes	-	IV (+3)	-	-	Yes (+144)	PVOD with pulmonary failure (+183)
	46	Female	7.8	Metachromatic leukodystrophy	UCB	UCB	1	BU + FLU + ATG	-	-	IV (+4)	Yes (+6)	-	-	-
	52	Male	14.6	Acute lymphoblastic leukemia	MUD	BM	1	TBI (12 Gy) + VP16 + ATG	-	-	IV (+5)	-	-	-	-
	68	Male	9.9	Acute lymphoblastic leukemia	MUD	BM	1	TBI (12 Gy) + VP16 + ATG	-	-	IV (0)	-	-	-	-
	69	Female	15.8	Acute myeloid leukemia	SIB	BM	1	BU + CY + MEL	Yes	-	IV (+8)	-	-	-	-
	75	Female	14.3	Acute myeloid leukemia	MUD	BM	1	BU + CY + MEL + ATG	Yes	-	IV (+4)	Yes (+13)	-	-	-
	80	Female	15.2	Acute myeloid leukemia	SIB	BM	1	BU + CY + MEL	Yes	-	IV (+8)	-	-	-	-
	86	Male	13.6	Chronic myeloid leukemia	MUD	BM	1	BU + CY + ATG	-	-	IV (+54)	-	-	-	Multi-organ failure (+111)
	1	Male	3.6	Acute lymphoblastic leukemia	MUD	PBSC	1	BU + CY + ATG	-	-	III (+11)	-	-	-	-
	5	Female	1.2	Hemophagocytic lymphohistiocytosis	MUD	BM	1	BU + CY + ATG	Yes	Yes (−9)	III (+5)	Yes (+12)	-	-	-
	11	Female	2.1	Acute lymphoblastic leukemia	UCB	UCB	1	BU + CY + MEL + ATG	Yes	-	III (+8)	-	-	-	-
	12	Female	11.6	Infantile osteopetrosis	SIB	BM	1	BU + FLU	Yes	-	III (+7)	-	-	-	-
	19	Male	5.1	Acute lymphoblastic leukemia	MUD	BM	1	TBI (12 Gy) + VP16 + ATG	-	-	III (+6)	-	-	-	-
	21	Male	14.3	Acute lymphoblastic leukemia	MUD	BM	1	TBI (12 Gy) + VP16 + ATG	Yes	-	III (+8)	-	-	-	-
	33	Male	5.7	Acute lymphoblastic leukemia	MMUD	BM	1	TBI (12 Gy) + VP16 + ATG	-	-	III (+7)	-	-	-	-
	48	Female	4.0	Acute myeloid leukemia	MMUD	BM	1	BU + CY + MEL + ATG	Yes	-	III (+43)	-	-	Yes (+43)	-
	56	Male	3.8	Kostmann agranulocytosis	MUD	PBSC	1	BU + FLU + ATG	-	Yes (−6)	III (+11)	-	-	-	-
	62	Female	8.0	Acute lymphoblastic leukemia	SIB	BM	1	BU + THIO + FLU	-	-	III (0)	Yes (+6)	-	Yes (+9)	-
	63	Female	7.9	Acute lymphoblastic leukemia	Haplo	PBSC	1	BU + THIO + FLU + ATG	-	-	III (+17)	Yes (+13)	-	-	-
	70	Female	2.9	Diamond-Blackfan anemia	MUD	BM	1	THIO + FLU + ATG	-	Yes (−7)	III (+7)	-	-	-	-
	77	Male	12.4	Acute lymphoblastic leukemia	MUD	BM	1	BU + THIO + FLU + ATG	-	-	III (+4)	-	-	-	-
	79	Female	15.2	Severe aplastic anemia	SIB	BM	1	CY + ATG	-	-	III (+2)	-	-	-	-
	82	Male	2.6	Juvenile chronic myeloid leukemia	SIB	BM	1	BU + CY + MEL	Yes	-	III (+1)	-	-	-	-
	84	Male	17.3	Acute lymphoblastic leukemia	MMUD	PBSC	1	TBI (12 Gy) + VP16 + ATG	Yes	-	III (0)	-	-	-	-
	85	Female	2.5	Acute lymphoblastic leukemia	SIB	UCB	1	BU + THIO + FLU	-	-	III (+33)	-	-	-	Relapse of leukemia (+174)
EBMT SOS grade I–II	16	Female	8.0	Acute lymphoblastic leukemia	SIB	BM	1	TBI (12 Gy) + VP16	-	-	II (+7)	-	-	-	-
	35	Male	2.2	Juvenile chronic myeloid leukemia	SIB	BM	1	BU + CY + MEL	Yes	-	II (+7)	-	-	-	-
	37	Female	3.3	Acute lymphoblastic leukemia	MUD	BM	1	BU + CY + VP16 + ATG	-	-	II (+10)	-	-	-	-
	38	Male	11.9	Severe combined immunodeficiency	MMUD	BM	1	BU + FLU + ATG	-	-	II (+11)	-	-	-	-
	57	Male	3.4	Acute myeloid leukemia	SIB	BM	1	BU + CY + MEL	Yes	-	II (+5)	-	-	-	-
	73	Female	7.6	Erythroblastic anemia	MUD	BM	1	THIO + FLU + ATG	-	-	II (+5)	-	-	-	-
	3	Female	3.6	Severe combined immunodeficiency	SIB	BM	2	FLU + TREO	Yes	-	I (+6)	-	-	-	-
	39	Male	2.0	Leukocyte adhesion deficiency	MUD	BM	1	FLU + TREO + ATG	-	-	I (+10)	-	-	-	-
	66	Female	13.4	Acute lymphoblastic leukemia	Haplo	PBSC	2	MEL + THIO + FLU + ATG	Yes	-	I (+5)	-	-	-	-
No EBMT SOS	2	Female	7.5	Acute myeloid leukemia	UCB	UCB	1	BU + CY + ATG	Yes	-	-	-	-	-	-
	4	Female	11.6	Acute lymphoblastic leukemia	MUD	BM	1	CY + VP16 + ATG	Yes	-	-	-	-	-	-
	6	Female	15.4	Blastic plasmacytoid dendritic cell leukemia	MUD	BM	1	TBI (12 Gy) + CY + ATG	-	-	-	-	-	-	-
	7	Male	13.1	Severe aplastic anemia	MUD	BM	1	CY + FLU + ATG	-	-	-	-	-	-	-
	8	Male	6.2	Acute lymphoblastic leukemia	MUD	BM	1	TBI (12 Gy) + VP16 + ATG	-	-	-	-	-	-	-
	9	Male	1.1	Acute lymphoblastic leukemia	MUD	BM	1	BU + CY + VP16 + ATG	-	-	-	-	-	-	Relapse of leukemia (+266)
	10	Male	5.8	Diffuse large cell lymphoma	SIB	BM	1	TBI (12 Gy) + VP16	-	-	-	-	-	-	Relapse of lymphoma (+82)
	13	Male	1.6	Hyper IgM syndrome	MUD	BM	1	BU + FLU + ATG	-	-	-	-	-	-	-
	14	Male	1.6	Hyper IgM syndrome	MUD	BM	1	BU + FLU + ATG	-	-	-	-	-	-	-
	15	Female	1.3	Hurler syndrome	MUD	BM	1	BU + CY + ATG	-	-	-	-	-	-	-
	18	Female	11.0	Myelodysplastic syndrome	MUD	BM	1	BU + CY + ATG	-	-	-	Yes (+4)	-	-	-
	20	Male	4.2	Hyper IgM syndrome	MUD	BM	1	BU + FLU + ATG	-	-	-	-	-	-	-
	22	Female	15.8	Duncans syndrome	MUD	BM	1	FLU + TREO + ATG	-	-	-	-	-	-	-
	23	Female	11.2	Severe aplastic anemia	MMUD	BM	1	TBI (2 Gy) + CY + ATG	-	-	-	-	-	-	-
	26	Female	1.4	Juvenile chronic myeloid leukemia	UCB	UCB	1	BU + CY + MEL + ATG	Yes	-	-	-	-	-	-
	27	Male	7.8	Severe aplastic anemia	MMUD	BM	1	TBI (2 Gy) + CY + ATG	-	-	-	-	-	-	-
	28	Male	10.2	Acute myeloid leukemia	MUD	BM	1	BU + CY + MEL + ATG	Yes	-	-	-	-	-	-
	29	Male	7.6	Severe aplastic anemia	MUD	BM	1	TBI (2 Gy) + CY + ATG	-	-	-	-	-	-	-
	30	Female	4.7	Myelodysplastic syndrome	MUD	BM	1	BU + CY + MEL + ATG	Yes	-	-	-	-	-	-
	31	Female	8.5	Myelodysplastic syndrome	MUD	BM	1	CY + FLU + ATG	-	-	-	-	-	-	-
	32	Male	8.3	Acute lymphoblastic leukemia	MUD	BM	1	TBI (12 Gy) + VP16 + ATG	-	-	-	-	-	-	-
	36	Male	12.0	Fanconi anemia	MUD	BM	1	CY + FLU + ATG	-	-	-	-	-	-	-
	40	Male	5.7	Acute lymphoblastic leukemia	MUD	BM	1	TBI (12 Gy) + VP16 + ATG	-	-	-	-	-	-	-
	42	Female	9.1	Sickle Thalassemia major	SIB	BM	1	THIO + FLU + ATG	-	-	-	-	-	-	-
	43	Male	4.4	Burkitts lymphoma	Haplo	PBSC	1	MEL + THIO + ATG	-	-	-	-	-	-	Relapse of lymphoma (+89)
	45	Male	16.6	Acute lymphoblastic leukemia	MUD	PBSC	1	TBI (12 Gy) + VP16 + ATG	-	-	-	-	-	-	-
	47	Female	7.1	Fanconi anemia	UCB	UCB	1	CY + FLU + ATG	-	-	-	-	-	-	-
	49	Male	5.7	Acute lymphoblastic leukemia	MUD	BM	1	TBI (12 Gy) + VP16 + ATG	-	-	-	-	-	-	-
	50	Male	13.0	SHOX syndrome	SIB	BM	1	FLU + TREO	-	-	-	-	-	-	-
	51	Female	7.3	Chronic granulomatous disease	MMUD	BM	1	BU + FLU + ATG	-	-	-	-	-	-	-
	53	Male	7.4	Acute lymphoblastic leukemia	SIB	BM	1	TBI (12 Gy) + VP16	-	-	-	-	-	-	-
	54	Male	3.2	Acute myeloid leukemia	MUD	BM	1	BU + CY + MEL + ATG	Yes	-	-	-	-	-	-
	55	Male	7.3	Acute lymphoblastic leukemia	SIB	BM	1	TBI (12 Gy) + VP16	-	-	-	-	-	-	-
	58	Female	12.8	Myelodysplastic syndrome	MUD	BM	1	THIO + FLU + ATG	-	-	-	-	-	-	-
	59	Female	13.9	Severe aplastic anemia	MUD	BM	1	TBI (2 Gy) + CY + ATG	-	-	-	-	-	-	-
	60	Male	6.9	Fanconi anemia	SIB	BM	1	CY + FLU + ATG	-	-	-	-	-	-	-
	61	Male	3.9	Acute lymphoblastic leukemia	Haplo	PBSC	1	BU + THIO + FLU + ATG	-	-	-	-	-	-	-
	64	Female	4.5	Congenital anemia	MUD	BM	1	THIO + FLU + ATG	-	-	-	-	-	-	-
	65	Female	1.5	Hemophagocytic lymphohistiocytosis	MUD	BM	1	FLU + TREO + ATG	Yes	Yes (−2)	-	-	-	-	-
	67	Male	2.9	X-linked lymphoproliferative syndrome	SIB	BM	1	BU + FLU	-	Yes (−8)	-	-	-	-	-
	71	Female	7.1	Large cell anaplastic lymphoma	SIB	BM	1	TBI (12 Gy) + VP16	-	-	-	-	-	-	-
	72	Male	16.4	Acute lymphoblastic leukemia	Haplo	PBSC	1	BU + THIO + FLU + ATG	-	-	-	-	-	-	-
	74	Female	10.6	Fanconi anemia	MMUD	BM	1	CY + FLU + ATG	-	-	-	-	-	-	-
	76	Male	14.4	Severe aplastic anemia	MMUD	BM	1	CY + FLU	-	-	-	-	-	-	-
	78	Male	8.2	Adrenoleukodystrophy	Haplo	PBSC	1	CY + THIO + FLU	Yes	-	-	-	-	-	Progression of disease (+241)
	81	Male	8.2	X-linked lymphoproliferative syndrome	UCB	UCB	1	BU + FLU + ATG	-	-	-	-	-	-	-
	83	Male	10.1	Sickle Thalassemia major	SIB	BM	1	THIO + FLU + ATG	-	-	-	-	-	-	-
	87	Female	16.1	Sickle Thalassemia major	SIB	BM	1	THIO + FLU + ATG	-	-	-	-	-	-	-

*SIB* matched sibling donor, *MUD* matched unrelated donor, *MMUD* mismatched unrelated donor, *UCB* umbilical cord blood, *Haplo* haploidentical donor, *BM* bone marrow, *PBSC* peripheral blood stem cells, *TBI* total body irradiation, *BU* busulfan, *CY* cyclophosphamide, *MEL* melphalan, *FLU* fludarabin, *Treo* treosulfan, *VP16* etoposide, *THIO* thiotepa, *PVOD* pulmonary veno-occlusive disease, *ALL* acute lymphoblastic anemia

Defibrotide (25 mg/kg/day) was given to five patients as prophylaxis due to diagnoses with increased risk of SOS (*n* = 3) or pre-existing liver disease (*n* = 2) with a median length of treatment of 35 days (31–40 days). Three patients on defibrotide prophylaxis developed grade III–IV SOS according to EBMT criteria.

### SOS and duration of primary admission

**Fig. 2:**
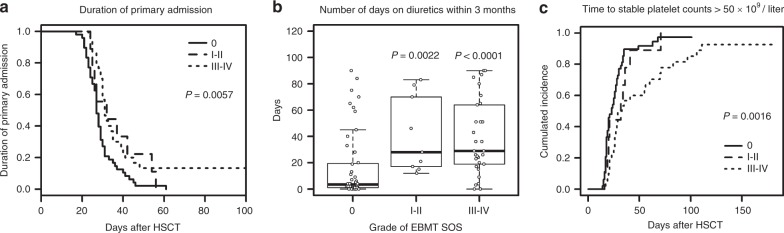
**a** Duration of primary admission after hematopoietic stem cell transplantation according to the severity grading of SOS based on pediatric EBMT criteria. Kaplan–Meier estimates with log-rank test for grade 0, grade I–II, and grade III–IV SOS are shown. The *p*-value indicates a significant difference between the three groups. Patients fulfilling grade III–IV SOS had a longer duration of their primary (*p* = 0.001) than patients without SOS. There was no significant difference between patients with milder degrees of SOS and patients with no SOS. **b** Number of days on diuretics within the first 3 months after HSCT for patients fulfilling grade 0, grade I–II, and grade III–IV SOS. The *p*-values indicate the difference between the groups of SOS patients and patients without SOS (using the Mann–Whitney-*U* test). **c** Time to stable platelet counts >50 × 10^9^/L for patients fulfilling grade 0, grade I–II, and grade III–IV SOS shown as Kaplan–Meier estimates with log-rank test. The *p*-value indicates a significant difference between the three groups. Stable platelet counts >50 × 10^9^/L were achieved later in patients with grade III–IV SOS compared to patients without SOS (*p* = 0.0003), while no significant difference was seen for patients with milder SOS

Next, we evaluated the course of HSCT in patients with SOS defined by the EBMT criteria in comparison with patients not fulfilling these criteria. Patients fulfilling grade III–IV SOS had a longer duration of their primary admission (31 days (23–183) vs. 27 days (17–61), *p* = 0.001) than patients without SOS. In contrast, the duration of stay in hospital did not differ between patients with milder degrees of SOS and patients with no SOS (Fig. 2a). The number of patients admitted to the ICU was too low (*n* = 3) for assessment of any association with the pediatric EBMT criteria.

### Use of diuretics

Patients fulfilling the pediatric EBMT SOS criteria received diuretics for more days post-HSCT within the first 3 months (29 days (0–90) vs. 3.5 days (0–90), *p* < 0.0001). These differences were significant both for grade III–IV SOS and grade I–II SOS compared with patients without SOS (*p* < 0.0001 and *p* = 0.0022, respectively) (Fig. 2b).

### Engraftment and GvHD

Time to neutrophil engraftment did not differ between patients with and without SOS. However, stable platelet counts >50 × 10^9^/L were achieved later in patients with grade III–IV SOS compared to patients without SOS (31 days (17–183) vs. 22 days (14–101), *p* = 0.0003), while no significant difference was seen for patients with milder SOS (Fig. 2c).

No difference was observed in risk of acute (*p* = 0.31) or chronic (*p* = 0.99) GvHD between patients fulfilling and not fulfilling EBMT SOS criteria (results not shown).

### Mortality

Seven out of 87 patients (8.0%) died within the first year following HSCT. One of the patients transplanted for non-malignant disorders died due to the progression of neurologic manifestations of metachromatic leukodystrophy post-HSCT. Six patients transplanted for malignant diseases relapsed, four of these with a fatal outcome.

**Fig. 3:**
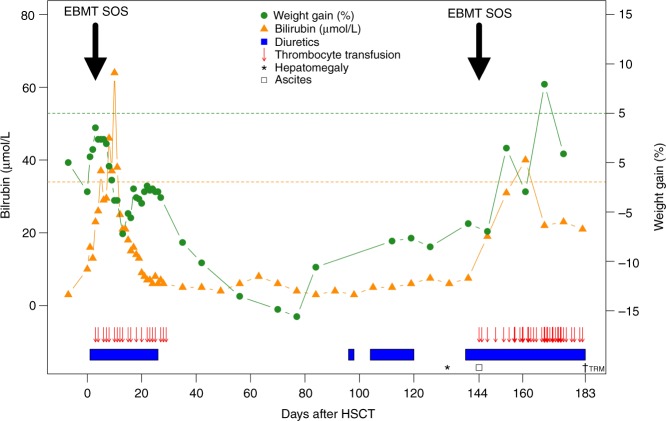
Patient with EBMT verified SOS not fulfilling Seattle/Baltimore criteria: HSCT course for an 8-year old girl with AML in second complete remission (CR2), transplanted with bone marrow from a matched unrelated donor after conditioning with intravenous PK-adjusted busulfan, cyclophosphamide, and melphalan. Clinical suspicion of SOS from day +5 post-HSCT due to a rise of bilirubin >34 µmol/L, however without fulfillment of other Seattle or Baltimore criteria (weight gain below 5% and no hepatomegaly/ascites/pain). By retrospective application of the EBMT SOS pediatric criteria, this patient fulfilled the criteria for grade IV SOS based on rapid and high increase in bilirubin, weight gain despite use of diuretics and unexplained consumptive RT. Moreover, she showed impaired coagulation (reduced coagulation factors and increased international normalized ratio), reduced kidney function (estimated glomerular filtration rate of 28 ml/min) and the need for continuous positive airway pressure (CPAP) for pulmonary ventilation. Around day +144, this patient developed rapidly rising bilirubin, UL-confirmed ascites, weight gain, consumptive RT, highly increased liver enzymes (ALT peaking at 2820 U/L), impaired coagulation, and the need for ventilator support, again selectively fulfilling the pediatric EBMT criteria of very severe SOS. In parallel, this patient developed respiratory insufficiency with pulmonary hypertension. At this stage, she was commenced on defibrotide, but died in multi-organ failure on day +183 post-HSCT. A post-mortem lung-biopsy showed changes suspicious for pulmonary VOD. The figure illustrates the various clinical parameters from day of HSCT (day 0) until transplant-related mortality (TRM). The dotted green line marks a weight gain of 5%. The dotted orange line marks bilirubin at 34 µmol/L. The time points for fulfillment of pediatric EBMT criteria are marked with black arrows

Two patients died in remission of treatment-related complications, both selectively fulfilling the pediatric EBMT criteria for very severe SOS, but neither fulfilling the classical criteria. One of these patients died in multiorgan failure day +111, initially dominated by liver failure, propagating to kidney failure, and respiratory insufficiency. The second patient developed progressing liver failure and respiratory insufficiency with signs of pulmonary hypertension 4.5 months after HSCT and passed away in a condition of multiorgan failure. A post-mortem lung-biopsy showed changes indicating pulmonary VOD (Fig. 3).

This limited frequency of mortality did not allow further statistical assessment in relation to fulfillment of EBMT SOS criteria.

## Discussion

The new diagnostic EBMT criteria for pediatric SOS were developed in an attempt to create a more dynamic diagnostic tool adapted to the pediatric characteristics of SOS. Since these criteria, like Seattle and Baltimore criteria, were developed based on expert opinion rather than clinical data, they warrant assessment and validation in clinical cohorts. Although retrospective in design, the overall results of this study indicate that these new criteria could be useful in the clinic and may help to identify patients with severe SOS and a poor outcome that do not fulfill Seattle or Baltimore criteria. Accordingly, the EBMT criteria appear to compensate for the shortcomings of Seattle and Baltimore criteria in the pediatric setting.

The modification to more dynamic assessment of weight gain and hyperbilirubinemia as well as the addition of the consumptive RT criterion was the primary cause that a larger proportion of patients in our cohort were diagnosed with SOS using the pediatric EBMT criteria. Although most children still developed hyperbilirubinemia during SOS, the alteration of the essential requirement of rising bilirubin in the EBMT criteria allowed for diagnoses of SOS in six patients with moderate/severe SOS despite the absence of hyperbilirubinemia.

The classical criteria restrict disease onset to 21 days post-HSCT, despite the fact that late occurring symptoms of SOS are frequent in children. The absence of this time restriction in the EBMT criteria played a minor role, being critical for only 3 patients, and fulfillment of hepatomegaly and ascites criteria was not critical for any patient in this study.

Potential advantages of the new pediatric EBMT criteria are related to minimization of imprecise and partly subjective clinical assessment of parameters such as pain, ascites, and hepatomegaly. This is done with the implementation of imaging, potentially increasing the reliability of the diagnosis. By applying baseline values, the new criteria also correct for shortcomings related to individual variations caused by pre-existing clinical conditions, which may be more frequent in the pediatric setting (e.g., immunodeficiencies).

However, there remain some challenges in the clinical implementation of the new EBMT criteria. *Rising bilirubin from a baseline value on 3 consecutive days* and *otherwise unexplained weight gain on 3 consecutive days despite the use of diuretics* are not fully defined regarding magnitude of the deviation from the normal. Further, guidelines as to whether thrombocytopenia should be interpreted as mainly consumptive or transfusion refractory could be more closely defined, although the EBMT severity grading criteria indicate that persistent RT >3 days is representative of moderate SOS. In this study, we have investigated different interpretations and chosen the most consistent assessment based on our patient data. We hope this can help give perspective and further optimize the criteria.

Application of the pediatric EBMT criteria in this study defined a broader group of patients diagnosed with SOS than the group defined by the Seattle criteria, though most of these patients are still included when using the pediatric EBMT criteria. The comparatively large number of patients fulfilling the pediatric EBMT criteria indicates a risk of over-diagnosis. There is, for instance, a risk that slight increases in bilirubin may be caused by hepatotoxic side effects of medication frequently used in the clinic, such as antibiotics, antiviral drugs and antifungals (in particular voriconazole [33] and carbapenems [34]). As we are the first to assess these proposed criteria, we have only our own numbers to consider regarding the risk of over-diagnosis.

However, when considering treatment of SOS based on the new EBMT criteria, we find that application of the severity grading criteria could possibly limit the group of patients where treatment is indicated. Overall, patients meeting the EBMT criteria for SOS had a longer duration of the primary admission, later occurrence of stable platelet counts, and received diuretics for a longer period, indicating higher morbidity. This was also the case for patients with grade III–IV SOS, but generally not for patients with grade I–II SOS. Importantly, application of the EBMT criteria also identified two patients among the very severe SOS group not identified by the Seattle criteria, who developed liver insufficiency progressing to fatal multiorgan failure. This indicates that patients fulfilling severe–very severe SOS have significantly higher morbidity and could benefit from earlier recognition of SOS and initiation of treatment, while mild–moderate SOS diagnosed by the EBMT criteria might not require treatment. The median time of diagnosis was rather early for our patients compared to the previously reported debut at around 2 weeks post-HSCT [7–9], however, this may be explained by our retrospective study design allowing for strict daily assessment compared to clinical observations.

Defibrotide was not implemented in our clinic in the beginning of the study period. Accordingly, only a few patients were treated with defibrotide on suspicion of SOS, and all these retrospectively fulfilled grade III–IV SOS by EBMT criteria. Thus, this study suggests an increased clinical awareness of patients fulfilling severe–very severe EBMT SOS criteria. Further optimization and adjustment of the pediatric EBMT SOS criteria should be based on prospective studies.

The main limitation of this study is the retrospective use of the EBMT criteria. The lack of patients fulfilling Baltimore criteria can partly be explained by improper clinical assessment and registration of hepatomegaly and ascites throughout this period, as well as the inaccuracy of this clinical investigation, especially in children. In general, there was an absence of baseline ultrasound for most of our patients as this has not been the practice in our clinic previously. However, none of our SOS diagnoses by the pediatric EBMT criteria were dependent on hepatomegaly/ascites alone and thus our results should not be altered. Further limitations are that competing conditions may be difficult to assess retrospectively as well as clinically, especially those that mimic SOS such as thrombotic microangiopathy and engraftment syndrome. In addition, the high survival rates in this study did not allow any conclusive analysis of mortality, indicating that the proposed EBMT criteria should be assessed in larger pediatric cohorts. The low frequency of high-risk patients in our study compared to others, as well as the limited use of TBI, could partly account for the generally excellent outcomes [9, 35]. However, this is difficult to assess due to variation and inconsistency in risk assessment of SOS in the literature as well as the specific inclusion of high-risk patients in many studies of pediatric SOS.

In conclusion, our findings suggest that application of the pediatric EBMT diagnostic and severity grading criteria for SOS may be helpful in identifying patients at increased risk of severe treatment-related complications and mortality. However, further assessment of the EBMT criteria based on larger prospective studies with the potential for clinical intervention is needed.
